# Comparison of Quantitative Structure-Activity Relationship Model Performances on Carboquinone Derivatives

**DOI:** 10.1100/tsw.2009.131

**Published:** 2009-10-14

**Authors:** Sorana D. Bolboaca, Lorentz Jäntschi

**Affiliations:** ^1^ Iuliu Hatieganu University of Medicine and Pharmacy Cluj-Napoca, Department of Medical Informatics and Biostatistics, 400349 Cluj-Napoca, Romania; ^2^ Technical University of Cluj-Napoca, 400641 Cluj-Napoca, Romania

**Keywords:** quantitative structure-activity relationship (qSAR), model validation, model assessment, Molecular Descriptors Family (MDF), Molecular Descriptors Family on Vertices (MDFV), carboquinone derivatives

## Abstract

Quantitative structure-activity relationship (qSAR) models are used to understand how the structure and activity of chemical compounds relate. In the present study, 37 carboquinone derivatives were evaluated and two different qSAR models were developed using members of the Molecular Descriptors Family (MDF) and the Molecular Descriptors Family on Vertices (MDFV). The usual parameters of regression models and the following estimators were defined and calculated in order to analyze the validity and to compare the models: Akaike?s information criteria (three parameters), Schwarz (or Bayesian) information criterion, Amemiya prediction criterion, Hannan-Quinn criterion, Kubinyi function, Steiger's Z test, and Akaike's weights. The MDF and MDFV models proved to have the same estimation ability of the goodness-of-fit according to Steiger's Z test. The MDFV model proved to be the best model for the considered carboquinone derivatives according to the defined information and prediction criteria, Kubinyi function, and Akaike's weights.

### INTRODUCTION

Quantitative structure-property/activity relationship (QSPR/qSAR) models may be considered data mining applications[1]. These methods are used to estimate/predict physical-chemical properties[2,3] and/or biological activities[4] of compounds, or to classify molecules[5] based on structural features. Besides their usefulness in compound screening[6], QSPR/qSAR models are also used due to their ability to explain action mechanics for the investigated compounds[7].

Natural and synthetic quinoid compounds are known to be biologically active compounds with antibacterial[8,9], antifungal[10,11], antiprotozoal[12,13], virus inhibitory[14], and antitumor activities[15,16]. The biological activity of quinoid compounds has been investigated by using structure-activity relationship approaches since 1969[17].

Carboquinone derivatives, a type of quinoid compound, were synthesized by Nakao et al.[18] and used as anticancer drugs. Yoshimoto et al.[19] identified a linear dependence between antileukemic activity and the hydrophobic constant of 2,5-bis(1-aziridinyl)-p-benzoquinone derivatives.

The antileukemic activity of carboquinones expressed as the minimum effective dose (MED) and the optimum effective dose (OED) was previously modeled using the electrotopological state and the molecular connectivity indices with multiple linear regression (MLR)[20]. A four-descriptor model was identified for MED ($R^2$ = 0.90 and s = 0.21; $R^2$ is the determination coefficient and s is standard error of estimate). The same model obtained also revealed the ability to estimate the OED ($R^2$ = 0.88, s = 0.19).

Srivastava and Khan showed in a qSAR study that –OH and $-NH_2$ groups had an important contribution to the biological activity as terminal substituents[21]. Kawakami et al.[22] used a self-organizing map to analyze qSARs on carboquinone derivatives. The identified model proved able to predict biological activity (MED) with an average of error equal to 4.2% (0.87 squared of cross-validation correlation coefficient). The relationship between the structure and activity of carboquinone derivatives was also investigated by using neural networks[23,24].

The main differences of the approaches applied in investigation of carboquinone derivatives consisted of the use of different methods to generate descriptors and/or to identify the descriptors better able to explain the activity of the compounds. In addition, models with improved statistical quality as compared with previously reported models on carboquinone derivatives were published; unfortunately, the significance of this improvement was not quantified.

Our research reports the results of the MED of carboquinone derivatives for the same molecular set studied by Kawakami et al.[22]. Two families of structural descriptors, the Molecular Descriptors Family (MDF) and the Molecular Descriptors Family on Vertices (MDFV), were used to generate descriptors. Forward stepwise regression was applied for descriptor selection. The models (MDF, MDFV, and the previously reported model[22]) were compared in order to identify the method with the highest performance.

### MATERIALS AND METHODS

#### Data Set: Carboquinone Derivatives

The inverse of molar concentration, expressed in logarithmic scale, was taken from previously published research[22]. Molar concentration is the MED per 1 kg of mice able to prolong life by 40% compared with controls (administration of a small-quantity dosage in chronic injection)[19]. The generic structure of the investigated compounds is presented in Fig. 1. The abbreviation of the compounds, the substituent, and the observed and estimated activities are presented in Table 1.

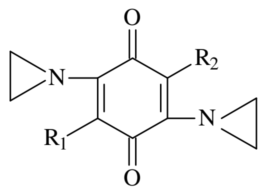

$R_1$ = …; $R_2$ = … (see Table 1)

**FIGURE 1**. Generic structure of carboquinone derivatives.

The observed activity of interest[22] was subject to statistical analysis in order to test the normality of data (assumption of multiple regression and condition for inference making). The observed activity had a mean of 5.76, a standard deviation of 0.63, a skewness of –0.12, and a kurtosis of 0.41. The Jarqua-Bera

**TABLE 1**
**Carboquinone Derivatives, Observed and Estimated Activities, and Residuals**

| Mol | $R_1$ Substituent | $R_2$ Substituent | Y | $\hat{Y}_{MDF}$ | $\hat{Y}_{MDFV}$ | $Res_{MDF}$ | $Res_{MDFV}$ |
|---|---|---|---|---|---|---|---|
| $cqd_{01}$ | $C_6H_5$ | $C_6H_5$ | 4.33 | 4.25 | 4.30 | 0.08 | 0.03 |
| $cqd_{02}$ | $CH_3$ | $(CH_2)_3C_6H_5$ | 4.47 | 4.71 | 4.34 | –0.24 | 0.13 |
| $cqd_{03}$ | $C_5H_{11}$ | $C_5H_{11}$ | 4.63 | 4.68 | 4.51 | –0.05 | 0.12 |
| $cqd_{04}$ | $CH(CH_3)_2$ | $CH(CH_3)_2$ | 4.77 | 4.85 | 4.89 | –0.08 | –0.12 |
| $cqd_{05}$ | $CH_3$ | $CH_2C_6H_5$ | 4.85 | 4.90 | 4.91 | –0.05 | –0.06 |
| $cqd_{06}$ | $C_3H_7$ | $C_3H_7$ | 4.92 | 4.88 | 4.90 | 0.04 | 0.02 |
| $cqd_{07}$ | $CH_3$ | $CH_2OC_6H_5$ | 5.15 | 5.29 | 5.28 | –0.14 | –0.13 |
| $cqd_{08}$ | $CH_2CH_2OCON(CH_3)_2$ | | 5.16 | 5.11 | 5.30 | 0.05 | –0.14 |
| $cqd_{09}$ | $C_2H_5$ | $C_2H_5$ | 5.46 | 5.25 | 5.52 | 0.21 | –0.06 |
| $cqd_{10}$ | $CH_3$ | $CH_2CH_2OCH_3$ | 5.57 | 5.94 | 5.59 | –0.37 | –0.02 |
| $cqd_{11}$ | $OCH_3$ | $OCH_3$ | 5.59 | 5.56 | 5.84 | 0.03 | –0.25 |
| $cqd_{12}$ | $CH_3$ | $CH(CH_3)_2$ | 5.60 | 5.75 | 5.48 | –0.15 | 0.12 |
| $cqd_{13}$ | $C_3H_7$ | $CH(OCH_3)CH_2OCONH_2$ | 5.63 | 5.80 | 5.86 | –0.17 | –0.23 |
| $cqd_{14}$ | $CH_3$ | $CH_3$ | 5.66 | 5.65 | 5.79 | 0.01 | –0.13 |
| $cqd_{15}$ | H | $CH(CH_3)_2$ | 5.68 | 5.81 | 5.79 | –0.13 | –0.11 |
| $cqd_{16}$ | $CH_3$ | $CH(OCH_3)C_2H_5$ | 5.68 | 5.60 | 5.73 | 0.08 | –0.05 |
| $cqd_{17}$ | $C_3H_7$ | $CH_2CH_2OCONH_2$ | 5.68 | 5.89 | 5.85 | –0.21 | –0.17 |
| $cqd_{18}$ | $CH_2CH_2OCH_3$ | | 5.69 | 5.63 | 5.43 | 0.06 | 0.26 |
| $cqd_{19}$ | $C_2H_5$ | $CH(OC_2H_5)CH_2OCONH_2$ | 5.76 | 5.68 | 5.87 | 0.08 | –0.11 |
| $cqd_{20}$ | $CH_3$ | $CH_2CH_2OCOCH_3$ | 5.78 | 6.02 | 5.67 | –0.24 | 0.11 |
| $cqd_{21}$ | $CH_3$ | $(CH_2)_3$-dimer | 5.82 | 5.58 | 5.64 | 0.24 | 0.18 |
| $cqd_{22}$ | $CH_3$ | $C_2H_5$ | 5.86 | 5.88 | 5.80 | –0.02 | 0.06 |
| $cqd_{23}$ | $CH_3$ | $CH(OCH_2CH_2OCH_3)^{-}$ | 6.03 | 5.63 | 6.03 | 0.40 | 0.00 |
| $cqd_{24}$ | $CH_3$ | $CH_2CH(CH_3)OCONH_2$ | 6.14 | 5.94 | 6.05 | 0.20 | 0.09 |
| $cqd_{25}$ | $C_2H_5$ | $CH(OCH_3)CH_2OCONH_2$ | 6.16 | 6.29 | 6.07 | –0.13 | 0.09 |
| $cqd_{26}$ | $CH_3$ | $CH(C_2H_5)CH_2OCONH_2$ | 6.18 | 6.01 | 6.23 | 0.17 | –0.05 |
| $cqd_{27}$ | $CH_3$ | $CH(OC_2H_5)CH_2OCONH_2$ | 6.18 | 5.95 | 6.26 | 0.23 | –0.08 |
| $cqd_{28}$ | $CH_3$ | $(CH_2)_3OCONH_2$ | 6.18 | 6.16 | 6.24 | 0.02 | –0.06 |
| $cqd_{29}$ | $CH_3$ | $(CH_2)_2OCONH_2$ | 6.21 | 6.43 | 6.31 | –0.22 | –0.10 |
| $cqd_{30}$ | $C_2H_5$ | $(CH_2)_2OCONH_2$ | 6.25 | 6.21 | 6.21 | 0.04 | 0.04 |
| $cqd_{31}$ | $CH_3$ | $CH_2CH_2OH$ | 6.39 | 6.50 | 6.29 | –0.11 | 0.10 |
| $cqd_{32}$ | $CH_3$ | $CH(CH_3)CH_2OCONH_2$ | 6.41 | 6.40 | 6.35 | 0.01 | 0.06 |
| $cqd_{33}$ | $CH_3$ | $CH(OCH_3)CH_2OCONH_2$ | 6.41 | 6.41 | 6.16 | 0.00 | 0.25 |
| $cqd_{34}$ | H | $N(CH_2)_2$ | 6.45 | 6.52 | 6.67 | –0.07 | –0.22 |
| $cqd_{35}$ | $CH_2CH_2OH$ | | 6.54 | 6.54 | 6.49 | 0.00 | 0.05 |
| $cqd_{36}$ | $CH_3$ | $N(CH_2)_2$ | 6.77 | 6.44 | 6.54 | 0.33 | 0.23 |
| $cqd_{37}$ | $CH_3$ | $CH(OCH_3)CH_2OH$ | 6.90 | 6.82 | 6.72 | 0.08 | 0.18 |
| JB (*p*) | | | | | | 0.3004(0.86) | 1.0054(0.60) |

Y = log(1/MED), where MED = minimum effective dose; $\hat{Y}_{MDF}$ = activity estimated by the MDF model; $\hat{Y}_{MDFV}$ = activity estimated by the MDFV model; $Res_{MDF}$ = residuals of the MDF model (Y – $\hat{Y}_{MDF}$); $Res_{MDFV}$ = residuals of the MDFV model (Y – $\hat{Y}_{MDFV}$); JB = Jarque-Bera value (*p*-value).

test[25] (two degrees of freedom) was applied to test the normality of observed data and a value of 1.66 was obtained ($p$ = 0.44). The Grubbs test[26] did not identify any outlier in the observed data (Grubbs value = 2.25 for the furthest data from the rest ($cqd_{01}$), $p > 0.05$).

The approach used to calculate molecular descriptors (MDF and MDFV) are detailed in the Appendix.

### Models Search, Validation, and Comparison

Multivariate regressions were obtained through systemic or random search for MDF and MDFV members by using client-server applications developed in Borland Delphi (v.6) and FreePascal (v.2). The task was performed after the filtration, identification, and removal of bias descriptors (as in the above-stated statistical validation of descriptors).

The best model obtained by each approach was selected according to the following criteria[33,34]:

- The highest explanation of the observed variance (highest values of significant correlation coefficients between the observed and estimated activity). A model was considered valid IF all correlation coefficients (Pearson (r), semi-Quantitative ($r_{sQ}$), Spearman (ρ), Kendall's ($\tau_a$, $\tau_b$, $\tau_c$), and Gamma (Γ)[35]) were statistically significant. The absence of at least one correlation coefficient that is not statistically significant leads to the exclusion of the model from further analysis.
- The smallest number of descriptors in the model.
- The lowest standard error of estimate ($s_{est}$).
- The highest Fisher value (the lowest $p$-value); significant coefficients of the regression model (highest t-value, lowest associated $p$-value).
- Internal validation: leave-one-out (loo) and leave-many-out (the sample was randomly divided into training and test sets, with ~62% compounds in the training set).
- External validation: a sample of 30 compounds with similar structures was used in order to predict the inverse of molar concentration. The compounds' abbreviation, $R_1$ and $R_2$ substituents were: $cqdt_{01}$ ($R_1$=H, $R_2$=H), $cqdt_{02}$ ($R_1$=H; $R_2=CH_3$), $cqdt_{03}$ ($R_1$=H, $R_2=C_3H_7$), $cqdt_{04}$ ($R_1$=H, $R_2=C_6H_5$), $cqdt_{05}$ ($R_1=CH_3$, $C_6H_5$), $cqdt_{06}$ ($R_1=C_2H_5$, $R_2=C_6H_5$), $cqdt_{07}$ ($R_1=OCH_3$, $R_2$=H), $cqdt_{08}$ ($R_1=OCH_3$, $R_2=CH_3$), $cqdt_{09}$ ($R_1=OCH_3$, $R_2=C_2H_5$), $cqdt_{10}$ ($R_1=C_5H_{11}$, $R_2$=H), $cqdt_{11}$ ($R_1=C_5H_{11}$, $R_2=CH_3$), $cqdt_{12}$ ($R_1=C_5H_{11}$, $R_2=OCH_3$), $cqdt_{13}$ ($R_1=CH_2CH_2OCON(CH_3)_2$, $R_2$=H), $cqdt_{14}$ ($R_1=CH_2CH_2OCON(CH_3)_2$, $R_2$=OH), $cqdt_{15}$ ($R_1=CH_2CH_2OCON(CH_3)_2$, $R_2=CH_3$), $cqdt_{16}$ ($R_1=CH_2CH_2OCON(CH_3)_2$, $R_2=OCH_3$), $cqdt_{17}$ ($R_1$=H, $R_2=CH_2C_6H_5$), $cqdt_{18}$ ($R_1$=H, $R_2=CH_2OC_6H_5$), $cqdt_{19}$ ($R_1=OCH_3$, $R_2=CH_2OC_6H_5$), $cqdt_{20}$ ($R_1=OCH_3$, $R_2=CH(CH_3)_2$), $cqdt_{21}$ ($R_1$=H, $R_2=CH(OCH_3)CH_2OCONH_2$), $cqdt_{22}$ ($R_1=CH_3$, $R_2=CH(OC_2H_5)CH_2OCONH_2$), $cqdt_{23}$ ($R_1$=H, $R_2=(CH_2)_3OCONH_2$), $cqdt_{24}$ ($R_1=C_2H_5$, $R_2=CH_2CH_2OH$), $cqdt_{25}$ ($R_1$=H, $R_2=CH(CH_3)CH_2OCONH_2$), $cqdt_{26}$ ($R_1=CH_3$, $R_2=CH_2CH_2OH$), $cqdt_{27}$ ($R_1$=H, $R_2=CH_2CH(CH_3)OCONH_2$), $cqdt_{28}$ ($R_1$=H, $R_2=CH(OC_2H_5)CH_2OCONH_2$), $cqdt_{29}$ ($R_1$=H, $R_2=CH(C_2H_5)CH_2OCONH_2$), $cqdt_{30}$ ($R_1$=H, $R_1=CH(OCH_3)CH_2OH$).
- The absence of collinearity between pairs of descriptors (correlation coefficient not statistically significant when applying all correlation methods (Pearson (r), semi-Quantitative ($r_{sQ}$), Spearman (ρ), Kendall's ($\tau_a$, $\tau_b$, $\tau_c$), and Gamma (Γ))[35].

The following parameters and/or tests were used as validation and comparison methods:

- Akaike information criteria (AIC[36]) and related formulas: consider the statistical goodness-of-fit and the number of parameters able to achieve the degree of fit. Its corrected formula ($AIC_c$)[37] proved to be a better model selection criterion[38] and was used in the study. The following related criteria were calculated to select the best models:

$$AIC_c = AIC + \frac{2k(k+1)}{n-k-1}, \quad AIC = n \cdot \ln\left(\frac{RSS}{n}\right) + 2k \tag{1}$$

$$AIC_{R^2} = \ln\left(\frac{1-R^2}{n}\right) + 2k \tag{2}$$

$$AIC_u = \ln\left(\frac{RSS}{n-k}\right) + \frac{n+k}{n-k-2} \text{ [39]} \tag{3}$$

$$BIC = n \cdot \ln\left(\frac{RSS}{n-k}\right) + k \cdot \ln(n) \text{ [40]} \tag{4}$$

$$APC = \frac{1}{n} \cdot RSS \cdot \frac{n+k}{n-k} \text{ [41]} \tag{5}$$

$$HQC = n \cdot \ln\left(\frac{RSS}{n}\right) + 2 \cdot k \cdot \ln(\ln(n)) \text{[42]} \tag{6}$$

where $AIC_c$ = corrected AIC for bias adjustment in small sample sizes (applied when the n/k ratio is below 40); $AIC_R{}^2$ = AIC based on the determination coefficient; $AIC_u$ = McQuarrie and Tsai corrected AIC; BIC = Schwarz (or Bayesian) Information Criterion (also abbreviated as SIC); APC = Amemiya Prediction Criterion; HQC = Hannan-Quinn Criterion; n = sample size; k = number of parameters in the model; RSS = residual sums of squares. The preferred model was the one with the lowest AIC, BIC, APC, and HQC values.

- Kubinyi function (FIT)[43,44]:

$$FIT = \frac{r^2 \cdot (n-k-1)}{(n+k^2) \cdot (1-r^2)} \tag{7}$$

The highest the FIT value, the better the model was considered.

- The best model is considered the one with the smallest relative distance from the "truth". The difference between the model with the lowest AIC and the others ($\Delta_i = AIC_i - \min(AIC)$, where $\Delta_i$ = difference between the AIC of the best fitting model and that of model *i*; $AIC_i$ = AIC corrected for model *i*; min(AIC) = minimum AIC value of all models). The formula used in this analysis was[45]:

$$w_i = \frac{\exp(-0.5 \cdot \Delta_i)}{\sum_{j=1}^{J} \exp\left(-0.5 \cdot \sum \Delta_j\right)} \tag{8}$$

where $w_i$ = Akaike weights for model *i*; denominator = sum of the relative likelihoods for all candidate models; j = number of models. The Akaike weights were calculated based on Eqs. 1–3.

- The comparison of correlation coefficients obtained by two models was performed by applying the Steiger's Z test at a significance level of 5%[46].

### RESULTS

The valid MDF and MDFV members on the carboquinone sample were included in the multivariate regression analysis in order to obtain qSAR models. One MDF (see Eq. 9) and one MDFV (see Eq. 11) model with the best performances were chosen from statistically significant models and are presented.

The estimated activity values associated to each model and the residuals are shown in Table 1. The values of descriptors used in the MDF and MDFV models are presented in Table 2.

**TABLE 2**
**Value of Descriptors in Eq. 9 - MDF and Eq. 11 - MDFV Models**

| Mol | MDF Model | | | | | MDFV Model | | | |
|---|---|---|---|---|---|---|---|---|---|
| | IGDMIQt | IbMDpHg | IHMmIHt | IHDDfHg | IHDMkMg | TEuIFFDL | GLClicdI | TAkaFcDL | GLbIAcDR |
| $cqd_{01}$ | 1.3039 | 28.2480 | 4.2884 | –0.0293 | 0.0120 | 0.3221 | 0.9851 | 2.1948 | 49.8200 |
| $cqd_{02}$ | 1.2571 | 66.1930 | 3.1964 | 0.1588 | 0.0103 | 0.1903 | 1.0000 | 2.2578 | 49.2500 |
| $cqd_{03}$ | 1.4600 | 45.6300 | 2.4894 | 0.4288 | 0.0096 | 0.1930 | 0.9826 | 2.3021 | 52.8100 |
| $cqd_{04}$ | 1.0610 | 18.9800 | 2.9734 | 0.2940 | 0.0097 | 0.1601 | 1.0000 | 1.2754 | 55.9100 |
| $cqd_{05}$ | 1.2756 | 42.2230 | 3.5552 | 0.3379 | 0.0110 | 0.1675 | 0.9824 | 1.9046 | 49.7600 |
| $cqd_{06}$ | 1.2283 | 23.0510 | 2.7513 | 0.3296 | 0.0095 | 0.1460 | 1.0000 | 1.3150 | 56.0100 |
| $cqd_{07}$ | 0.9357 | 52.9570 | 3.5809 | 0.1927 | 0.0104 | 0.1696 | 0.9824 | 1.6696 | 40.7500 |
| $cqd_{08}$ | 0.5203 | 98.9590 | 3.3759 | –0.0926 | 0.0096 | 0.0806 | 1.0000 | 2.3848 | 17.7280 |
| $cqd_{09}$ | 1.2777 | 17.6600 | 2.9248 | 0.2014 | 0.0093 | 0.0812 | 0.9826 | 1.0246 | 56.8800 |
| $cqd_{10}$ | 0.8050 | 32.9120 | 2.9853 | 0.3440 | 0.0091 | 0.0345 | 1.0000 | 1.1547 | 43.1100 |
| $cqd_{11}$ | 0.6325 | 19.1350 | 3.3724 | –0.1191 | 0.0089 | 0.0503 | 1.0000 | 1.0720 | 33.6700 |
| $cqd_{12}$ | 1.0405 | 19.5930 | 3.0140 | 0.3482 | 0.0093 | 0.0720 | 0.9826 | 1.0749 | 57.7400 |
| $cqd_{13}$ | 0.5569 | 41.7360 | 3.2895 | 0.1741 | 0.0093 | –0.0512 | 0.9671 | 2.0179 | 39.7800 |
| $cqd_{14}$ | 1.0264 | 14.0400 | 3.1212 | –0.0544 | 0.0088 | –0.0045 | 0.9824 | 0.8108 | 59.7600 |
| $cqd_{15}$ | 0.9692 | 19.2230 | 2.9352 | 0.3747 | 0.0091 | 0.0086 | 0.9826 | 0.7947 | 59.0300 |
| $cqd_{16}$ | 0.7632 | 32.7620 | 3.0629 | 0.5313 | 0.0097 | 0.1216 | 0.9826 | 1.0919 | 42.1800 |
| $cqd_{17}$ | 0.6846 | 45.8930 | 3.0854 | 0.0382 | 0.0087 | –0.1179 | 0.9877 | 1.6973 | 41.1500 |
| $cqd_{18}$ | 0.6738 | 37.9210 | 2.9662 | 0.2223 | 0.0089 | 0.0911 | 1.0000 | 1.5281 | 34.0100 |
| $cqd_{19}$ | 0.5620 | 53.8050 | 3.3005 | 0.1628 | 0.0094 | –0.0405 | 0.9671 | 1.9086 | 41.4200 |
| $cqd_{20}$ | 0.6732 | 47.7630 | 3.1697 | 0.0068 | 0.0087 | –0.1422 | 0.9978 | 1.7685 | 42.1500 |
| $cqd_{21}$ | 1.1294 | 24.0580 | 2.8746 | 0.3510 | 0.0093 | 0.0658 | 0.9826 | 0.8301 | 58.3100 |
| $cqd_{22}$ | 1.1489 | 17.1590 | 3.0050 | 0.2474 | 0.0091 | 0.0345 | 0.9826 | 0.6881 | 58.7500 |
| $cqd_{23}$ | 0.5596 | 58.0630 | 2.9227 | 0.1843 | 0.0088 | –0.0244 | 0.9589 | 1.7888 | 42.2200 |
| $cqd_{24}$ | 0.6035 | 51.6520 | 3.2512 | 0.0539 | 0.0090 | –0.1048 | 0.9721 | 1.8220 | 39.1000 |
| $cqd_{25}$ | 0.5573 | 53.6170 | 3.3661 | 0.1698 | 0.0092 | –0.0704 | 0.9721 | 1.7677 | 36.5000 |
| $cqd_{26}$ | 0.7349 | 57.0170 | 3.2272 | 0.1060 | 0.0091 | –0.0795 | 0.9721 | 1.3575 | 41.7600 |
| $cqd_{27}$ | 0.5064 | 59.1150 | 3.3366 | 0.1115 | 0.0092 | –0.0613 | 0.9721 | 1.4279 | 37.0900 |
| $cqd_{28}$ | 0.6165 | 62.7490 | 3.0616 | –0.1600 | 0.0082 | –0.1709 | 0.9794 | 1.4822 | 42.1400 |
| $cqd_{29}$ | 0.6054 | 50.1440 | 3.2157 | –0.0251 | 0.0085 | –0.1614 | 0.9877 | 1.1223 | 42.1600 |
| $cqd_{30}$ | 0.6805 | 48.1450 | 3.1626 | 0.0355 | 0.0087 | –0.1384 | 0.9877 | 1.2224 | 41.4000 |
| $cqd_{31}$ | 0.8915 | 24.3500 | 3.0163 | 0.3015 | 0.0087 | –0.1777 | 0.9826 | 1.0843 | 48.9500 |
| $cqd_{32}$ | 0.6645 | 52.7040 | 3.2735 | 0.0742 | 0.0088 | –0.1159 | 0.9721 | 1.3030 | 41.9500 |
| $cqd_{33}$ | 0.4947 | 55.5150 | 3.4174 | 0.0743 | 0.0090 | –0.0918 | 0.9721 | 1.6847 | 37.0900 |
| $cqd_{34}$ | 1.2019 | 16.4120 | 3.1159 | 0.3460 | 0.0091 | 0.0004 | 0.9626 | 0.5827 | 43.1400 |
| $cqd_{35}$ | 0.7980 | 24.3720 | 2.9806 | 0.2010 | 0.0084 | –0.1305 | 0.9826 | 1.1679 | 34.1000 |
| $cqd_{36}$ | 1.2712 | 17.4170 | 3.1845 | 0.3174 | 0.0093 | 0.0643 | 0.9625 | 0.5645 | 42.7100 |
| $cqd_{37}$ | 1.0000 | 30.5710 | 3.1890 | 0.4815 | 0.0093 | –0.0685 | 0.9824 | 1.0919 | 20.6680 |

The following MDF model performed best:

$$
\begin{aligned}
\hat{Y}_{MDF} = {} & 10.18(\pm 0.858) + IGDMlQt \cdot 1.03(\pm 0.446) + \\
& + IbMDpHg \cdot 0.01(\pm 0.005) + IHMmlHt \cdot 2.99(\pm 0.475) + \\
& + lHDDfHg \cdot 3.12(\pm 0.568) - IHDMkMg \cdot 1694(\pm 219) \\
& R^2 = 0.9305; s_{est} = 0.18; n = 37; F_{est}\ (p) = 83\ (5.26 \cdot 10^{-17}); \\
& t_{int}\ (p) = 24.20\ (1.09 \cdot 10^{-21});\ t_{X_1}\ (p) = 4.73\ (4.66 \cdot 10^{-5}); \\
& t_{X_2}\ (p) = 3.91\ (4.75 \cdot 10^{-4});\ t_{X_3}\ (p) = 12.82\ (6.30 \cdot 10^{-14}); \\
& t_{X_4}\ (p) = 11.20\ (2.00 \cdot 10^{-12});\ t_{X_5}\ (p) = -15.78\ (2.29 \cdot 10^{-16}); \\
& R^2_{loo} = 0.9057; s_{loo} = 0.21; F_{loo}\ (p) = 59\ (6.11 \cdot 10^{-15}) \\
& r\ (p) = 0.9646\ (7.56 \cdot 10^{-22}); r_{sQ}\ (p) = 0.9459\ (1.09 \cdot 10^{-18}) \\
& \rho\ (p) = 0.9276\ (1.55 \cdot 10^{-16}); \tau_a\ (p) = 0.7943\ (4.56 \cdot 10^{-12}); \\
& \tau_b\ (p) = 0.7943\ (4.56 \cdot 10^{-12}); \tau_c\ (p) = 0.7728\ (1.68 \cdot 10^{-11}) \\
& \Gamma\ (p) = 0.8027\ (1.99 \cdot 10^{-8})
\end{aligned}
\qquad (9)
$$

where $\hat{Y}_{MDF}$ = activity estimated by MDF model; IGDMlQt ($X_1$), IbMDpHg ($X_2$), IHMmlHt ($X_3$), lHDDfHg ($X_4$), and IHDMkMg ($X_5$) = MDF members; the values from round brackets allows us to obtain the lowest (subtraction) and upper (addition) confidence boundary for the slope parameters; $R^2$ = determination coefficient; $s_{est}$ = standard error of estimate; n = sample size; $F_{est}$ (p) = Fisher value of the MDF model (*p*-value); t = t-value; int = intercept; p = *p*-value;$R^2_{loo}$ = cross-validation leave-one-out square correlation coefficient; $s_{loo}$ = standard error of predicted; $F_{loo}$ = Fisher value on cross-validation leave-one-out model; r = Pearson correlation coefficient between observed activity and estimated by the model; $r_{sQ}$ = semi-quantitative correlation coefficient; ρ = Spearman rank correlation coefficient; $\tau_a$, $\tau_b$, $\tau_c$ = Kendall's correlation coefficients; Γ = Gamma correlation coefficient.

The MDF descriptors in Eq. 9 did not significantly correlate with the observed activity or between them when all correlation coefficients were investigated (see Table 3).

**TABLE 3**
**Matrix of Overall Correlation and Probability: MDF Model**

| | IbMDpHg | IHMmlHt | IHDDfHg | IHDMkMg | Y |
|---|---|---|---|---|---|
| IGDMlQt | × | × | × | × | × |
| IbMDpHg | ✓ | × | × | × | × |
| IHMmlHt | | ✓ | × | × | × |
| IHDDfHg | | | ✓ | × | × |
| IHDMkMg | | | | ✓ | × |
| Y | | | | | ✓ |

IGDMlQt, IbMDpHg, IHMmlHt, lHDDfHg, and IHDMkMg = MDF members – Eq. 9; Y = observed activity; × = not significant correlation (Pearson and semi-quantitative and Spearman and Kendall's and Gamma); ✓ = significant correlation (Pearson and semi-quantitative and Spearman and Kendall's and Gamma); significance level: 5%.

The results obtained in the leave-many-out analysis of the MDF model (Eq. 9) are:

$$\begin{aligned}
&\hat{Y}_{MDF-training} = 9.96(\pm 1.10) + IGDMlQt\cdot 1.41(\pm 0.72) + \\
&\quad + IbMDpHg\cdot 0.01(\pm 0.008) + IHMmlHt\cdot 3.06(\pm 0.83) + \\
&\quad + lHDDfHg\cdot 3.08(\pm 0.80) - IHDMkMg\cdot 1737(\pm 344) \\
&R^2_{tr} = 0.9614; s_{tr} = 0.19; n_{tr} = 23; F_{tr}\ (p) = 41\ (6.18\cdot 10^{-9}); \\
&t_{int}\ (p) = 19.13\ (6.17\cdot 10^{-13});\ t_{X_1}\ (p) = 4.15\ (6.67\cdot 10^{-4}); \\
&t_{X_2}\ (p) = 2.78\ (1.29\cdot 10^{-2});\ t_{X_3}\ (p) = 7.81\ (5.04\cdot 10^{-7}); \\
&t_{X_4}\ (p) = 8.11\ (3.01\cdot 10^{-7});\ t_{X_5}\ (p) = -10.65\ (6.09\cdot 10^{-9}); \\
&R^2_{ts} = 0.9191; n_{ts} = 14; F_{ts}\ (p) = 14\ (8.95\cdot 10^{-4})
\end{aligned} \qquad (10)$$

where $R^2_{tr}$ = determination coefficient of model obtained in the training set; $s_{tr}$ = standard error of estimate in training set; $n_{tr}$ = number of compounds in training set; $F_{tr}$ = Fisher value associated to the model obtained in training set; $R^2_{ts}$ = determination coefficient of model obtained in the test set; $s_{ts}$ = standard error of estimate in the test set; $n_{ts}$ = number of compounds in test set; $F_{tr}$ = Fisher value associated to the model obtained in test set. The following compounds were randomly included in the training set: $cqd_{02}$, $cqd_{03}$, $cqd_{04}$, $cqd_{06}$, $cqd_{12}$, $cqd_{13}$, $cqd_{14}$, $cqd_{18}$, $cqd_{19}$, $cqd_{23}$, $cqd_{26}$, $cqd_{27}$, $cqd_{28}$, and $cqd_{32}$.

The MDFV model that performed best and its characteristics are presented in Eq. 11:

$$\begin{aligned}
&\hat{Y}_{MDFV} = 24.26(\pm 4.324) - TEuIFFDL\cdot 2.40(\pm 0.469) - \\
&\quad - GLCIicdI\cdot 16.78(\pm 4.375) - TAkaFcDL\cdot 0.65(\pm 0.111) - \\
&\quad - GLbIAcDR\cdot 0.02(\pm 0.006) \\
&R^2 = 0.9548; s_{est} = 0.14; n = 37; F_{est}\ (p) = 169\ (5.00\cdot 10^{-21}); \\
&t_{int}\ (p) = 11.43\ (7.84\cdot 10^{-13});\ t_{X_1}\ (p) = -10.44\ (7.77\cdot 10^{-12}); \\
&t_{X_2}\ (p) = -7.81\ (6.52\cdot 10^{-9});\ t_{X_3}\ (p) = -11.94\ (2.52\cdot 10^{-13}); \\
&t_{X_4}\ (p) = -8.68\ (6.50\cdot 10^{-10}); \\
&R^2_{loo} = 0.9351; s_{loo} = 0.17; F_{loo}\ (p) = 115\ (5.41\cdot 10^{-20}); \\
&r = 0.9771\ (p = 4.04\cdot 10^{-25}); r_{sQ} = 0.9615\ (3.25\cdot 10^{-21}); \\
&\rho = 0.9461\ (1.03\cdot 10^{-18}); \tau_a = 0.8273\ (5.74\cdot 10^{13}); \\
&\tau_b = 0.8273\ (5.74\cdot 10^{-13}); \tau_c = 0.8050\ (2.35\cdot 10^{-12}); \\
&\Gamma = 0.8361\ (1.13\cdot 10^{-9})
\end{aligned} \qquad (11)$$

where $\hat{Y}_{MDFV}$ = the activity estimated by the MDFV model; TEuIFFDL ($X_1$), GLCIicdI ($X_2$), TAkaFcDL ($X_3$), and GLbIAcDR ($X_4$) = MDFV members.

The MDFV descriptors in Eq. 11 did not correlate significantly with the observed activity or between them when all the correlation coefficients were investigated (see Table 4).

**TABLE 4**
**Matrix of Overall Correlation and Probability: MDFV Model**

| | GLClicdl | TAkaFcDL | GLblAcDR | Y |
|---|---|---|---|---|
| TEuIFFDL | × | × | × | × |
| GLClicdl | ✓ | × | × | × |
| TAkaFcDL | | ✓ | × | × |
| GLblAcDR | | | ✓ | × |
| Y | | | | ✓ |

TEuIFFDL, GLClicdl, TAkaFcDL, and GLblAcDR = MDFV members – Eq. 11; Y = observed activity; × = not significant correlation (Pearson and semi-quantitative and Spearman and Kendall's and Gamma); ✓ = significant correlation (Pearson and semi-quantitative and Spearman and Kendall's and Gamma); significance level: 5%

The results obtained in leave-many-out analysis of MDFV model (Eq. 11) are:

$$\begin{aligned}
&\hat{Y}_{MDFV-training} = 25.48(\pm 5.816) - TEuIFFDL\cdot 2.45(\pm 0.6886) - \\
&\quad - GLClicdI\cdot 18.29(\pm 6.018) - TAkaFcDL\cdot 0.61(\pm 0.165) - \\
&\quad - GLbIAcDR\cdot 0.02(\pm 0.008) \\
&t_{int}\,(p) = 9.24\;(2.95\cdot 10^{-8});\;\; t_{X_1}\,(p) = -7.52\;(5.82\cdot 10^{-7}); \\
&t_{X_2}\,(p) = -6.41\;(4.91\cdot 10^{-6});\;\; t_{X_3}\,(p) = -7.79\;(3.60\cdot 10^{-7}); \\
&t_{X_4}\,(p) = -5.28\;(5.07\cdot 10^{-5}); \\
&R^2_{tr} = 0.9483; s_{tr} = 0.15; n_{tr} = 23; F_{tr}\,(p) = 83\;(2.50\cdot 10^{-11}); \\
&R^2_{ts} = 0.9659; s_{ts} = 0.17; n_{ts} = 14; F_{ts}\,(p) = 38\;(1.26\cdot 10^{-5})
\end{aligned} \tag{12}$$

The molecules included in the test set were: $cqd_{01}$, $cqd_{03}$, $cqd_{09}$, $cqd_{18}$, $cqd_{19}$, $cqd_{21}$, $cqd_{23}$, $cqd_{24}$, $cqd_{25}$, $cqd_{28}$, $cqd_{30}$, $cqd_{31}$, $cqd_{35}$, and $cqd_{37}$.

The values obtained by applying the validation and comparison parameters (Eqs. 1–8) for MDF and MDFV, as well as for the linear regression model obtained by using the previously reported descriptors (molar refractivity of the steric effects of $R_1$ and $R_2$, hydrophobicity of the steric effects of $R_1$ and $R_2$, hydrophobicity of the steric effect of $R_2$, molar refractivity of the steric effect of $R_1$, and two substituent's constants)[22], are shown in Table 5.

The goodness-of-fit of the MDF and MDFV models is presented in Figure 2.

The results of the Steiger's Z test are presented in Table 6.

An external set of compounds was used in order to predict the inverse of molar concentration using the best identified model (Eq. 11). The values of the descriptors and the predicted inverse of molar concentration (logarithmic scale) are presented in Table 7.

**TABLE 5**
**Validation and Comparison of the Models: Results of Parameters from Eqs. 1–8**

| **Parameter** | **Model** | | |
|---|---|---|---|
| | **MDF** | **MDFV** | **MLR[22]** |
| $AIC_c$ (corrected Akaike information criterion) | –118.58 | –137.35 | –92.65 |
| $w_i(AIC_c)$ | $1.33\cdot10^{-6}$ | $1.58\cdot10^{-2}$ | $3.10\cdot10^{-12}$ |
| $AIC_{R2}$ (AIC based on determination coefficient) | 5.72 | 3.29 | 4.50 |
| $w_i(AIC_{R2})$ | 0.16 | 0.54 | 0.30 |
| $AIC_u$ (McQuarrie and Tsai corrected AIC) | –1.95 | –2.49 | –1.28 |
| $w_i(AIC_u)$ | 0.33 | 0.43 | 0.24 |
| BIC (Schwarz, or Bayesian, Information Criterion) | –105.17 | –125.86 | –81.16 |
| APC (Amemiya prediction criterion) | 0.04 | 0.02 | 0.08 |
| HQC (Hannan-Quinn Criterion) | –117.97 | –136.44 | –91.74 |
| FIT (Kubinyi function) | 5.50 | 10.55 | 2.80 |

$w_i$ = Akaike weights for model *I*; Parameters: smallest the better excepting FIT and $w_i$ (where largest the better); MLR[22] = regression model obtained from the previously reported physical-chemical descriptors.

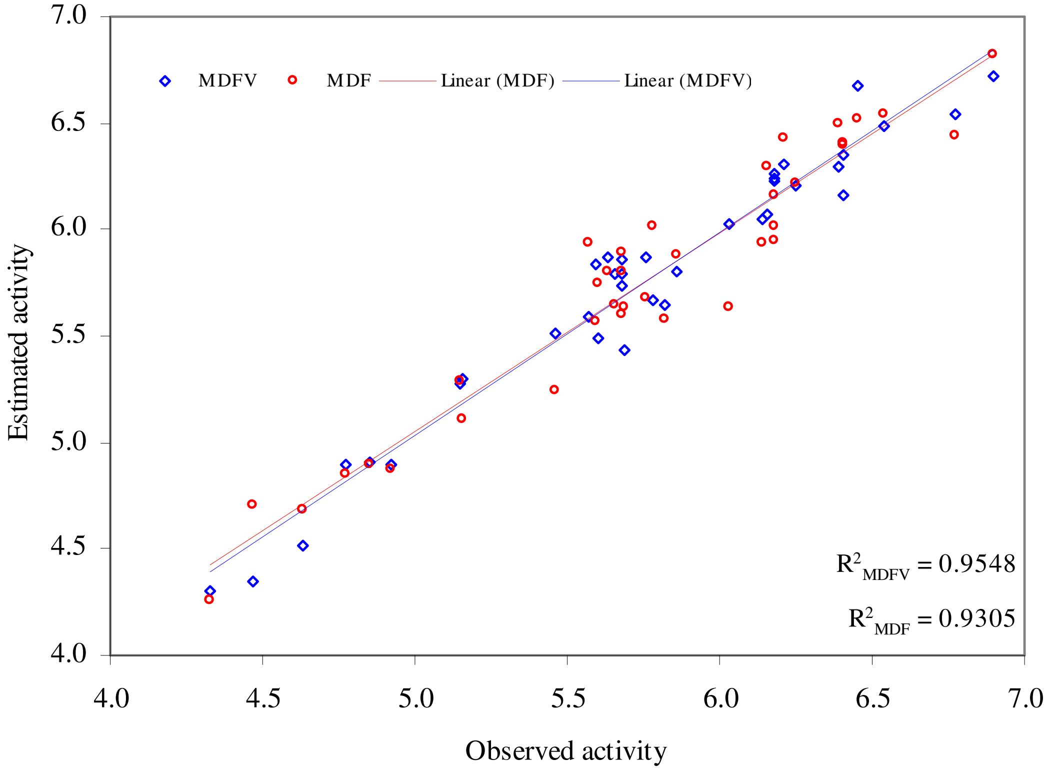

**FIGURE 2**. Goodness-of-fit of models: MDFV vs. MDF.

**TABLE 6**
**Results of Comparisons: Steiger's Test (Degree of Freedom = 34)**

| Parameter | MDFV vs. MDF | Parameter | MDFV vs. MLR[22] | Parameter | MDF vs. MLR[22] |
|---|---|---|---|---|---|
| $R_{Y\text{-}\hat{Y}MDFV}$ | 0.9771 | $R_{Y\text{-}\hat{Y}MDFV}$ | 0.9771 | $R_{Y\text{-}\hat{Y}MDF}$ | 0.9646 |
| $R_{Y\text{-}\hat{Y}MDF}$ | 0.9646 | $R_{Y\text{-}\hat{Y}MLR[22]}$ | 0.9212 | $R_{Y\text{-}\hat{Y}MLR[22]}$ | 0.9212 |
| $R_{\hat{Y}MDFV\text{-}\hat{Y}MDF}$ | 0.9514 | $R_{\hat{Y}MDFV\text{-}\hat{Y}MLR[22]}$ | 0.9414 | $R_{\hat{Y}MDF\text{-}\hat{Y}MLR[22]}$ | 0.9170 |
| $Z_{Steiger}$ | 1.28 | $Z_{Steiger}$ | 3.95 | $Z_{Steiger}$ | 2.40 |
| *p* | 0.1008 | *p* | $3.90 \cdot 10^{-5}$ | *p* | $8.15 \cdot 10^{-3}$ |

Y = log(1/MED), where MED = minimum effective dose; $\hat{Y}_{MDF}$ = activity estimated by the MDF model; $\hat{Y}_{MDFV}$ = activity estimated by the MDFV model; MLR[22] = regression model obtained from the previously reported physical-chemical descriptors; R = correlation coefficient; $Z_{Steiger}$ = Steiger Z value; *p* = *p*-value.

**TABLE 7**
**Inverse of Molar Concentration: Predicted Values on the External Set**

| Mol | TEuIFFDL | GLClicdI | TAkaFcDL | GLbIAcDR | $\hat{Y}_{MDFV\text{-}Eq11}$ |
|---|---|---|---|---|---|
| cqdt01 | –0.1278 | 1.0000 | 0.4121 | 63.5900 | 5.9617 |
| cqdt02 | –0.0731 | 0.9827 | 0.4773 | 34.2300 | 6.7970 |
| cqdt03 | 0.0021 | 0.9821 | 0.9261 | 31.3860 | 6.4030 |
| cqdt04 | 0.0980 | 0.9828 | 1.1826 | 32.9600 | 5.9547 |
| cqdt05 | 0.1561 | 0.9671 | 1.5260 | 32.8900 | 5.8560 |
| cqdt06 | 0.1975 | 1.0000 | 1.5388 | 30.4620 | 5.2553 |
| cqdt07 | –0.0461 | 1.0000 | 0.5626 | 25.9570 | 6.5886 |
| cqdt08 | 0.0207 | 0.9830 | 0.6819 | 25.9280 | 6.6362 |
| cqdt09 | 0.0636 | 0.9823 | 0.8200 | 24.1860 | 6.4972 |
| cqdt10 | 0.0536 | 0.9823 | 1.5006 | 31.0060 | 5.9099 |
| cqdt11 | 0.1093 | 1.0000 | 1.4937 | 30.9150 | 5.4857 |
| cqdt12 | 0.1261 | 0.9888 | 1.4530 | 22.3490 | 5.8696 |
| cqdt13 | –0.0398 | 0.9817 | 1.4184 | 24.5120 | 6.3573 |
| cqdt14 | –0.0236 | 1.0000 | 1.8202 | 18.5220 | 5.8956 |
| cqdt15 | –0.0172 | 1.0000 | 1.8202 | 22.8600 | 5.7739 |
| cqdt16 | –0.0035 | 1.0000 | 1.9078 | 18.4760 | 5.7913 |
| cqdt17 | 0.1101 | 0.9821 | 1.1830 | 29.5150 | 6.0214 |
| cqdt18 | 0.1123 | 0.9823 | 1.3496 | 24.5080 | 6.0267 |
| cqdt19 | 0.1951 | 1.0000 | 2.1987 | 18.5300 | 5.1226 |
| cqdt20 | 0.1001 | 0.9822 | 1.1181 | 23.6130 | 6.2306 |
| cqdt21 | –0.1162 | 0.9769 | 1.6657 | 21.8890 | 6.5243 |
| cqdt22 | –0.0613 | 0.9767 | 1.7191 | 22.1560 | 6.3542 |
| cqdt23 | –0.1947 | 0.8919 | 1.2641 | 19.9550 | 8.4488 |
| cqdt24 | –0.1486 | 0.9704 | 0.9920 | 23.8120 | 7.1039 |
| cqdt25 | –0.1409 | 0.8500 | 1.6342 | 22.1550 | 8.7273 |
| cqdt26 | –0.1777 | 1.0000 | 0.8301 | 24.6490 | 6.7623 |
| cqdt27 | –0.1296 | 0.9535 | 1.0798 | 13.7090 | 7.5319 |
| cqdt28 | –0.0850 | 0.8551 | 1.1519 | 22.3290 | 8.8179 |
| cqdt29 | –0.1036 | 0.8500 | 1.7019 | 22.5490 | 8.5838 |
| cqdt30 | –0.0992 | 0.9824 | 0.9055 | 24.4450 | 6.8249 |

### DISCUSSION

Three qSAR models were investigated in order to assess their ability to estimate the antileukemic activity of a sample of 37 carboquinone derivatives. Two approaches were used to calculate the molecular descriptors for the carboquinone derivatives: MDF and MDFV. The MDF approach proved able to estimate properties and activities[47,48,49,50,51,52]. The MDFV is a new approach that implements the fragmentation of vertices on the molecular graph. A similar approach on vertex cut proved its usefulness on b-ary trees[53]. The third analyzed qSAR model was obtained by using the physical-chemical descriptors reported by Kawakami et al.[22]. A series of classical and newly defined parameters were computed (Eqs. 1–8) in order to compare the models.

The qSAR models were selected according to the Hawkins principles[54]. The models with the highest correlation coefficient, the highest Fisher parameter, the lowest standard error of estimate, and the smallest possible number of significant parameters were chosen.

The MDF and MDFV models proved to have estimation abilities, demonstrated by the presence of statistically significant correlation coefficients between the observed and estimated activity (see Eq. 9 for the MDF model and Eq. 11 for the MDFV model).

The analysis of the MDF (Eq. 9) and MDFV (Eq. 11) models in terms of the descriptor's contribution to the activity of carboquinone derivatives revealed the following:

| qSAR model | Eq. 9 | Eq. 11 |
|---|---|---|
| qSAR determination (%) | 93% | 95% |
| Interaction via | Space (geometry - *g*) and<br>Bonds (topology - *t*) | Space (geometry - *G*) and<br>Bonds (topology - *T*) |
| Dominant atomic property | Charge (*Q*) and<br>Number of directly bonded hydrogen (*H*) and<br>Relative atomic mass (*M*) | Electronegativity (*E*) and<br>Melting point (*L*)<br>Electronic affinity (*A*) |
| Overlapping interaction | Frequent and distant interactions (*M, m*) and<br>Frequent and closed interactions (*D*) | Not applicable |
| Structure on activity scale | Identity (*I*) and<br>Logarithm of absolute value (*l*) | Logarithm (*L*) and<br>Identity (*I*) and<br>Reciprocal (*R*) |

The investigated activity of the carboquinone derivatives proved to be of geometric and topological nature. It depended on compound charge, number of directly bonded hydrogen atoms, and relative atomic mass in the MDF model (see Eq. 9) and on compound electronegativity, melting point, and electronic affinity in the MDFV model (see Eq. 11).

The absence of collinearity between the descriptors used by the MDF and MDFV models (see Tables 3 and 4), and the parameters obtained in leave-one-out and leave-many-out analyses (see Eqs. 10 and 12) supported the validity of these models.

As far as the comparison of models is concerned, a series of parameters were computed in order to identify the best qSAR model for carboquinone derivatives (see Table 5). The analysis of parameters presented in Table 5 leads to the following observations:

- The MDFV model (Eq. 11) systematically obtained the best expected values: the smallest value of prediction criteria ($AIC_c$, $AIC_{R2}$, $AIC_u$, BIC, APC, and HQC); the highest values of Akaike's weights ($w_i(AIC_c)$, $w_i(AIC_{R2})$, $w_i(AIC_u)$) and of the Kubinyi function (FIT).
- The overall classification of models in descending order of their performances according to all the parameters (Eqs. 1–8) is: MDFV – MDF – regression model obtained from the previously reported physical-chemical descriptors[22].

- In most cases, the MDF model registered the second performance. Two exceptions were observed: the model had the third performance according to the $AIC_{R2}$ and $w_i(AIC_{R2})$ criteria.
- The lowest value of BIC obtained for the MDFV model implied fewer descriptors and a better fit when the model was compared to the MDF model. It implied only better goodness-of-fit when compared with the model obtained from the previously reported physical-chemical descriptors[22].

The analysis of the results presented in Table 1 revealed that the mean of the observed and estimated activity are equal, but the standard deviation of activity estimated by MDF and MDFV models were slightly lower (a difference of 0.01 for MDFV model and of 0.02 for MDF model) compared to the standard error of observed data[22]. This observation leads to the existence of a possible risk of overprediction and could be assigned to random or systematic experimental errors. The intrinsic variability of experimental measurements pulls over the intrinsic variability of the model. If the experimental measurements are not valid, the model is not valid. The Jarque-Bera test[25] was applied on the observed data in order to investigate their normality and membership to the same population, as a measure for minimizing the overprediction (also a condition for MLR). The experimental data proved to be normally distributed and no outlier was identified by the Gubber test, even if the value of the furthest compound from the rest was include into the analysis.

As far as the goodness-of-fit of the MDF and MDFV models according to Steiger's Z test was concerned, these two models were not statistically different (see Table 6). The MDF and MDFV models proved to have significantly higher correlation coefficients compared to the regression model obtained from the previously reported physical-chemical descriptors[22] (see Table 6, $p < 0.01$).

The MDFV model was considered as the best model (considering the number of descriptors and the information criteria). Thus, this model was applied on an external sample of 30 compounds in order to predict the inverse of molar concentration (logarithmic scale). The values of the descriptors (see Table 7) had the same order of size and the average value of two descriptors proved to be covered into the 95% confidence interval of the descriptors' value in sample of 37 compounds. The predicted values of the inverse of molar concentration expressed in logarithmic scale showed the highest values (more potent compounds) compared to the sample of 37 compounds. The standard deviation is also a little bit higher as well as the average of predicted values. Note that the predicted values need to be experimentally validated in order to sustain the potency of these compounds, the absence of this validation being the main limitation of the present study.

The present study aimed to compare three qSAR models in order to understand the relationship between the structure of the investigated carboquinone derivatives and the MED expressed in logarithmic scale. Two models were obtained by applying the MDF and MDFV approaches, while the third model was obtained from the physical-chemical descriptors reported by Kawakami et al.[22]. Useful information related to the structural nature of the investigated activity of carboquinone derivatives was obtained once the MDF and MDFV models were constructed. While the MDF approach has already proved its estimation and prediction potential[44,45,46,47,48,49], current research in our laboratory aims to characterize other activities and/or other chemical compounds in order to test the usefulness of fragmentation on vertices in the investigation of structure-activity relationships.

The statistical parameters of the MDF and MDFV models supported their validity. The MDF and MDFV models were not significantly different. Both models proved to have better goodness-of-fit compared with the model obtained from the previously reported physical-chemical descriptors[22]. The MDFV model proved to be the best model for the studied carboquinone derivatives according to the prediction criteria, and to the value of Kubinyi function.

The modeling process in qSARs is widely used by computational chemists, but unfortunately, different models obtained on the same class of compounds are not usually compared. The research used a series of information parameters besides the Steiger's Z test in order to assess and compare different qSAR models. The proposed concept was evaluated on a set of carboquinone derivatives. Future research is required in order to develop guidelines for comparing different qSAR models.

The SAR modeling using the MDFV approach gives an advantage due to its construction; a systematic pool of unique descriptors (the same descriptors with the same values are obtained any time when the approach is applied on the same structures) is obtained from the structure of a given set of compounds using two extreme (minimal and maximal) and three intermediate (harmonic, geometric, and arithmetic) operations, which are able to cumulate the physical contribution of the atoms to the activity of compounds. A small part of the descriptors explains (correlate) the activity/property based on structural information in a sample of compounds. The explanation power of the SAR model increases by embedding as much information as possible, as was proved in the text (the goodness-of-fit of the MDFV model presented in Eq. 11 is higher compared with the goodness-of-fit obtained in the training set model presented in Eq. 12). Thus, the described approach should be conducted by using as much information as possible in order to construct the relationships between the compound structure and activity/property (model), and the prediction should be limited to similar compounds (similar with the ones in the training set) as was conducted in this study. Using the proposed approach, the prediction of antileukemic activity was performed on a sample of compounds (the structure of the used compounds was similar to the structure of the compounds used to obtain the MDFV model). Note that the experimental value of the compounds included in the external validation set could not be found in the specialty literature using the available resources. Even if the results obtained in the internal validation of the MDFV model lead to good results, the predicted antileukemic activity needs to be correlated with experimental data and could lead to more active carboquinone derivatives with antileukemic activity.

### CONCLUSIONS

The MDF and MDFV approaches provided reliable and valid models in terms of statistical characterization, collinearity, leave-one-out and leave-many-out analyses. The MDF and MDFV models proved equally able to estimate the activity of carboquinone derivatives according to Steiger's Z test. The MDFV model proved to be the best model for the considered carboquinone derivatives according to the information and prediction criteria, Kubinyi function, and Akaike's weights.

### ACKNOWLEDGMENT

Financial support is gratefully acknowledged to UEFISCSU Romania (ID0458/206/01.10.2007).

## APPENDIX

### Molecular Descriptors Calculation

Two approaches were used to calculate the molecular descriptors for the sample of carboquinone derivatives: Molecular Descriptors Family (MDF)[27] and Molecular Descriptors Family on Vertices (MDFV). Both approaches integrate the complex topological and geometrical information obtained from the structure of the compounds by computing the family of descriptors used to explain the activity of interest.

The topological and geometrical models of the compounds were the input data in the investigation of carboquinone derivatives. The three-dimensional structures were drawn by using HyperChem version 7.01[28]. The compounds partial charges were calculated by using the semi-empirical extended Hückel model[29]. The geometry of compounds was optimized by applying the Austin method (AM1)[30]. The **.hin* files were the input molecular files and the **.txt* file was the input activity file used by both methods in order to generate and calculate the pools of descriptors. A brief description of the MDF and MDFV methods are presented below.

#### *Molecular Descriptors Family*

- Method principle: candidate fragments obtained using pairs of vertices.
- Physical model of interaction: for a pair of atoms.
- Physical model of atomic overlapping interaction: ▪ in fragments; ▪ cumulated for pairs of atoms; ▪ cumulated for entire molecule.
- Molecular topology: matrix representation of the molecular graphs.
- Name of MDF members: seven letters indicating how they were generated. The meanings of the letters are as follows:
  - 1st letter (linearization operator): identity (*I*), inverse (*i*), absolute value (*A*), inverse of absolute value (*a*), logarithm (*L*), logarithm of absolute value (*l*).
  - 2nd letter (global overlapping of fragments interaction): one value from the following four groups: ▪ *group of values*: minimum value (*m*), maximum value (*M*), lowest absolute value (*n*), highest absolute value (*N*); ▪ *group of means*: sum (*S*), arithmetic mean according to the number of fragment properties (*A*), arithmetic mean according to the number of fragments (*a*), arithmetic mean according to the number of atoms (*B*), arithmetic mean according to the number of bonds (*b*); ▪ *geometric group*: multiplication (*P*), geometric mean according to the number of fragment properties (*G*), geometric mean according to the number of fragments (*g*), geometric mean according to the number of atoms (*F*), geometric mean according to the number of bonds (*f*); ▪ *harmonic group*: harmonic sum (*s*), harmonic mean according to the number of fragment properties (*H*), harmonic mean according to the number of fragments (*h*), harmonic mean according to the number of atoms (*I*), harmonic mean according to the number of bonds (*i*).
  - 3rd letter (algorithm of molecular fragmentation applied on atomic pairs): fragmentation based on paths (Cluj[31]) (*P*) or on distances (Szeged[32]) (*D*); fragmentation in maximal fragments (*M*) or in minimal fragments (*m*).
  - 4th letter (overlapping interactions): models with sporadic and distant interactions (*R*, *r*), models with frequent and distant interactions (*M*, *m*), models with frequent and closed interactions (*D*, *d*).
  - 5th letter (interaction descriptor): could take one of the following values: $D=d$, $d=1/d$, $O=p_1$, $o=1/p_1$, $P=p_1p_2$, $p=1/p_1p_2$, $Q=\sqrt{p_1p_2}$, $q=1/\sqrt{(p_1p_2)}$, $J=p_1d$, $j=1/p_1d$, $K=p_1p_2d$, $k=1/p_1p_2d$,

$L=d\sqrt{(p_1p_2)}$, $l=1/d\sqrt{(p_1p_2)}$, $V=p_1/d$, $E=p_1/d_2$, $W=p_1^2/d$, $w=p_1p_2/d$, $F=p_1^2/d^2$, $f=p_1p_2/d^2$, $S=p_1^2/d^3$, $s=p_1p_2/d^3$, $T=p_1^2/d^4$, $t=p_1p_2/d^4$; where $d$ = distance operator and $p$ = atomic property.
- 6th letter (atomic property): relative atomic mass (*M*), atomic partial charge, semi-empirical extended Hückel model, single point approach (*Q*), cardinality (*C*), atomic electronegativity (*E*), group electronegativity (*G*), number of hydrogen atoms adjacent to the investigated atom (*H*).
- 7th letter (distance operator): geometric distance (*g*), topological distance (*t*).

- Statistical validation of MDF descriptors:
  - Determination coefficient calculated between value of descriptor and observed activity significantly differs from zero (significance level of $10^{-5}$).
  - The same rule was applied to determination coefficients obtained in pairs of descriptors.

### Molecular Descriptors Family on Vertices

- Method principle: candidate fragments obtained using cutting atoms (vertices cut).
- Physical model of interaction: for a pair of atoms.
- Physical model of atomic overlapping interaction: ▪ in fragments; ▪ cumulated for each atom; ▪ cumulated for entire molecule.
- Molecular topology: matrix representation of the molecular graphs.
- Name of MDFV member: eight letters indicating how they were generated. The meanings of the letters are as follows:
  - 1st letter (distance operator): geometric distance (*G*), topological distance (*T*).
  - 2nd letter (atomic property): all atomic properties implemented in MDF (except for the group electronegativity (*G*)) plus melting point under normal temperature and pressure conditions (*L*) and electronic affinity (*A*).
  - 3rd letter (interaction descriptor): $J=D$, $j=1/D$, $O=P_1$, $o=1/P_1$, $P=P_2$, $p=1/P_2$, $Q=P_1P_2$, $q=1/P_1P_2$, $R=\sqrt{(P_1P_2)}$, $r=1/\sqrt{(P_1P_2)}$, $K=P_1D$, $k=(1/P_1)D$, $L=P_2D$, $l=(1/P_2)D$, $M=P_1P_2D$, $m=(1/P_1P_2)D$, $N=\sqrt{(P_1P_2)}D$, $n=(1/\sqrt{(P_1P_2)})D$, $W=P_1D^2$, $w=(1/P_1)D^2$, $X=P_2D^2$, $x=(1/P_2)D^2$, $Y=P_1P_2D^2$, $y=(1/P_1P_2)D^2$, $Z=\sqrt{(P_1P_2)}D^2$, $z=(1/\sqrt{(P_1P_2)})D^2$, $S=P_1/D$, $s=(1/P_1)/D$, $T=P_2/D$, $t=(1/P_2)/D$, $U=P_1P_2/D$, $u=(1/P_1P_2)/D$, $V=\sqrt{(P_1P_2)}/D$, $v=(1/\sqrt{(P_1P_2)})/D$, $F=P_1/D^2$, $f=(1/P_1)/D^2$, $G=P_2/D^2$, $g=(1/P_2)/D^2$, $H=P_1P_2/D^2$, $h=(1/P_1P_2)/D^2$, $I=\sqrt{(P_1P_2)}/D^2$, $i=(1/\sqrt{(P_1P_2)})/D^2$, $A=P_1/D^3$, $a=(1/P_1)/D^3$, $B=P_2/D^3$, $b=(1/P_2)/D^3$, $C=P_1P_2/D^3$, $c=(1/P_1P_2)/D^3$, $D=\sqrt{(P_1P_2)}/D^3$, $d=(1/\sqrt{(P_1P_2)})/D^3$, $0=P_1/D^4$, $1=(1/P_1)/D^4$, $2=P_2/D^4$, $3=(1/P_2)/D^4$, $4=P_1P_2/D^4$, $5=(1/P_1P_2)/D^4$, $6=\sqrt{(P_1P_2)}/D^4$, $7=(1/\sqrt{(P_1P_2)})/D^4$, where $D$ = distance operator and $P$ = atomic property.
  - 4th letter (overlapping interactions at fragment/vertices level) and 5th letter (overlapping interactions at molecule level): maximum value (*A*), maximum value of the sum of squares on the X, Z, and Y projections (*a*), minimum value (*I*), minimum value of the sum of squares on the X, Z, and Y projections (*i*), projection overlaps on axes (*F*), mediate the unity value of the descriptor on the X, Z, or Y projections and overlap the descriptors values (*P*), aggregate value in the center of descriptor (*C*).
  - 6th letter (interaction for each overlap and per atom/fragment): vectorial overlap of descriptors per fragment (*f*), vectorial overlap of descriptor per atom (*F*), aggregate in the center of descriptor per fragment (*c*), aggregate in the center of descriptor per atom (*C*), mediates the unity value of the descriptor on the X, Z, or Y projections and overlaps the descriptors values per fragments (*p*), mediates the unity value of the descriptor on the X, Z, or Y projections and overlaps the descriptors values per atom (*P*), absolute maximum value of descriptors - interactions in the fragment (*a*), absolute maximum value of descriptors - interaction of the fragment with the atom (*A*), absolute minimum value of descriptors - interactions in the fragment (*i*), absolute minimum value of descriptors - interaction of the fragment with the atom (*I*).

- 7th letter (expression units): value of molecular descriptor (*D*), value of the descriptor projection on the X, Z, and Y axes (*d*).
- 8th letter (linearization operator): identity (*I*), reciprocal (*R*), logarithm (*L*).

- Statistical validation of MDFV descriptors:
  - Delete all descriptors with a Jarque-Bera value higher than critical value for the observed activity[25].
  - Delete all descriptors with an intercorrelation higher than 0.99.

The molecular descriptors were calculated by using a series of PHP programs, run on an IntraNet network on a FreeBSD server. The applications used MySQL dynamic libraries to connect to MDF and MDFV databases where the descriptors and identified models were stored.
